# Lotharmeyerite, Ca(Zn,Mn)_2_(AsO_4_)_2_(H_2_O,OH)_2_

**DOI:** 10.1107/S1600536811054286

**Published:** 2011-12-23

**Authors:** Yongbo W. Yang, Stanley H. Evans, Robert T. Downs, Hexiong Yang

**Affiliations:** a Department of Chemsitry and Biochemistry, University of Arizona, 1306 E. University Blvd., Tucson, Arizona 85721-0041, USA; b Department of Geosciences, University of Arizona, 1040 E. 4th Street, Tucson, Arizona 85721-0077, USA

## Abstract

Lotharmeyerite, calcium bis­(zinc/manganese) bis­(arsenate) bis­(hydroxide/hydrate), Ca(Zn,Mn^3+^)_2_(AsO_4_)_2_(H_2_O,OH)_2_, is a member of the natrochalcite group of minerals, which are characterized by the general formula *AM*_2_(*X*O_4_)_2_(H_2_O,OH)_2_, where *A* may be occupied by Pb^2+^, Ca^2+^, Na^+^, and Bi^3+^, *M* by Fe^3+^, Mn^3+^, Cu^2+^, Zn^2+^, Co^2+^, Ni^2+^, Al^3+^, and Mg^2+^, and *X* by P^V^, As^V^, V^V^, and S^VI^. The minerals in the group display either monoclinic or triclinic symmetry, depending on the ordering of chemical components in the *M* site. Based on single-crystal X-ray diffraction data of a sample from the type locality, Mapimi, Durango, Mexico, this study presents the first structure determination of lotharmeyerite. Lotharmeyerite is isostructural with natrochalcite and tsumcorite. The structure is composed of rutile-type chains of edge-shared *M*O_6_ octa­hedra (site symmetry 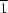) extending along [010], which are inter­connected by *X*O_4_ tetra­hedra (site symmetry 2) and hydrogen bonds to form [*M*_2_(*X*O_4_)_2_(OH,H_2_O)_2_] sheets parallel to (001). These sheets are linked by the larger *A* cations (site symmetry 2/*m*), as well as by hydrogen bonds. Bond-valence sums for the *M* cation, calculated with the parameters for Mn^3+^ and Mn^2+^ are 2.72 and 2.94 v.u., respectively, consistent with the occupation of the *M* site by Mn^3+^. Two distinct hydrogen bonds are present, one with O⋯O = 2.610 (4) Å and the other O⋯O = 2.595 (3) Å. One of the H-atom positions is disordered over two sites with 50% occupancy, in agreement with observations for other natrochalcite-type minerals, such as natrochalcite and tsumcorite.

## Related literature

For lotharmeyerite, see: Dunn (1983 ▶); Kampf *et al.* (1984 ▶); Brugger *et al.* (2002 ▶). For related minerals in the natrochalcite group, see: Tillmanns & Gebert (1973 ▶); Chevrier *et al.* (1993 ▶); Ansell *et al.* (1992 ▶); Krause *et al.* (1998 ▶, 1999 ▶, 2001 ▶); Brugger *et al.* (2000 ▶, 2002 ▶). Parameters for bond-valence calculations were taken from Brese & O’Keeffe (1991 ▶). For additional information on related minerals, see: Ferraris & Ivaldi (1984 ▶); Krickl & Wildner (2007 ▶).

## Experimental

### 

#### Crystal data

Ca(Zn·Mn)_2_(AsO_4_)_2_(H_2_O·OH)_2_
*M* *_r_* = 474.14
Monoclinic, 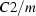

*a* = 9.0727 (6) Å
*b* = 6.2530 (4) Å
*c* = 7.4150 (5) Å
β = 116.739 (4)°
*V* = 375.68 (4) Å^3^
*Z* = 2
Mo *K*α radiation
μ = 14.38 mm^−1^
*T* = 293 K
0.06 × 0.05 × 0.05 mm

#### Data collection

Bruker APEXII CCD area-detector diffractometer
Absorption correction: multi-scan [*SADABS* (Sheldrick, 2005 ▶) and *XABS2* (Parkin *et al.*, 1995 ▶)] *T*_min_ = 0.477, *T*_max_ = 0.532
2512 measured reflections
739 independent reflections
659 reflections with *I* > 2σ(*I*)
*R*_int_ = 0.022

#### Refinement

*R*[*F* ^2^ > 2σ(*F* ^2^)] = 0.019
*wR*(*F* ^2^) = 0.045
*S* = 0.91
739 reflections
49 parameters
All H-atom parameters refined
Δρ_max_ = 0.81 e Å^−3^
Δρ_min_ = −0.77 e Å^−3^

### 

Data collection: *APEX2* (Bruker, 2004 ▶); cell refinement: *SAINT* (Bruker, 2004 ▶); data reduction: *SAINT*; program(s) used to solve structure: *SHELXS97* (Sheldrick, 2008 ▶); program(s) used to refine structure: *SHELXL97* (Sheldrick, 2008 ▶); molecular graphics: *XtalDraw* (Downs & Hall-Wallace, 2003 ▶); software used to prepare material for publication: *publCIF* (Westrip, 2010 ▶).

## Supplementary Material

Crystal structure: contains datablock(s) I, global. DOI: 10.1107/S1600536811054286/pk2375sup1.cif

Structure factors: contains datablock(s) I. DOI: 10.1107/S1600536811054286/pk2375Isup2.hkl

Additional supplementary materials:  crystallographic information; 3D view; checkCIF report

## Figures and Tables

**Table 1:** Hydrogen-bond geometry (Å, °)

*D*—H⋯*A*	*D*—H	H⋯*A*	*D*⋯*A*	*D*—H⋯*A*
O1—H1⋯O1^i^	0.82 (7)	1.79 (8)	2.610 (4)	177 (11)
O1—H2⋯O4^ii^	0.66 (5)	1.95 (5)	2.595 (3)	163 (6)

Symmetry codes: (i) 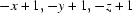; (ii) 
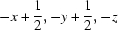
.

**Fig. 1.**
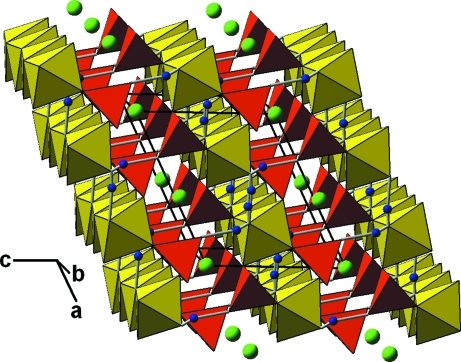
Crystal structure of lotharmeyerite. The large green and small blue spheres represent Ca and H atoms, respectively. The yellow octahedra and red tetrahedra represent MO4(H2O,OH)2 and AsO4 groups.

## supplementary crystallographic information

### Comment

The natrochalcite group of minerals is characterized by the general formula *AM*_2_(*X*O_4_)_2_(H_2_O,OH)_2_, where currently it is observed that *A* = Pb, Ca, Na, and Bi, *M* = Fe^3+^, Mn^3+^, Cu^2+^, Zn^2+^, Co^2+^, Ni^2+^, Al^3+^, and Mg^2+^, and *X* = P^5+^, As^5+^, V^5+^, and S^6+^ (Krause *et al.*, 1998, 2001; Brugger *et al.*, 2000; 2002). The majority of minerals in this group crystallize in monoclinic *C*2/*m* symmetry and a few in triclinic *P*1 symmetry. In particular, monoclinic natrochalcite-group minerals with *A* = Ca and *X* = As can be further assigned to the lotharmeyerite subgroup, which includes six members: lotharmeyerite (*M* = Zn) (Dunn, 1983; Kampf *et al.*, 1984; Brugger *et al.*, 2002), ferrilotharmeyerite (*M* = Fe^3+^) (Ansell *et al.*, 1992; Krause *et al.*, 1998), cobaltlotharmeyerite (*M* = Co) (Krause *et al.*, 1999), nickellotharmeyerite (*M* = Ni) (Krause *et al.*, 2001), manganlotharmeyerite (*M* = Mn^3+^) (Brugger *et al.*, 2002), and cabalzarite (*M* = Mg) (Brugger *et al.*, 2000).

Lotharmeyerite from the Ojuela mine, Mapimi, Mexico was first described by Dunn (1983) with the chemical formula CaZnMn^3+^(AsO_4_)_2_(OH).2H_2_O. This formula, however, was revised to CaZnMn^3+^(AsO_3_OH)_2_(OH)_3_ by Kampf *et al.* (1984) on the basis of the infrared spectroscopic data measured on the specimen from the type locality. Unfortunately, due to the very small crystals in drusy growths, which gave somewhat diffuse and split spots on precession films, Kampf *et al.* (1984) only obtained the unit-cell parameters for this mineral: *a* = 9.066 (4), *b* = 6.276 (2), *c* = 7.408 (2) Å, β = 116.16 (3)°, V = 378.3 (4) Å^3^. From the structure refinement of ferrilotharmeyerite, the Fe^3+^ analogue of lotharmeyerite, Krause *et al.* (1998) proposed a new chemical formula for lotharmeyerite as Ca(Mn^3+^,Zn)_2_(AsO_4_)_2_(OH,H_2_O)_2_. Yet, by defining lotharmeyerite and manganlotharmeyerite to represent the Zn- and Mn-dominant endmembers of the lotharmeyerite subgroup, Brugger *et al.* (2002) made another revision of the chemical formula of lotharmeyerite to Ca(Zn,Mn^3+^)_2_(AsO_4_)_2_(H_2_O,OH)_2_ based on a new chemical analysis from a lotharmeyerite crystal from the type locality. Thus far, the crystal structures of all minerals, except lotharmeyerite, in the lotharmeyerite subgroup have been determined. This study reports the first structure refinement of lotharmeyerite from the type locality by means of single-crystal X-ray diffraction data.

Lotharmeyerite is isotypic with other monoclinic natrochalcite-group minerals (*e.g.*, Tillmanns & Gebert, 1973; Chevrier *et al.*, 1993; Krause *et al.*, 1998, 1999, 2001; Brugger *et al.*, 2000; 2002). Its structure is composed of rutile-type chains of edge-shared *M*O_6_ octahedra extending along [010], which are interconnected by *X*O_4_ tetrahedra and hydrogen bonds to form [*M*_2_(*X*O_4_)_2_(OH,H_2_O)_2_] sheets parallel to (001) (Fig. 1). These sheets are linked together by the larger *A* cations, as well as hydrogen bonds. Bond-valence sums for the *M* cation, calculated with the parameters for Mn^3+^ and Mn^2+^ (Brese & O'Keeffe, 1991), are 2.72 and 2.94 v.u., respectively, consistent with the occupation of the *M* site by Mn^3+^ (Brugger *et al.* 2002).

The presence of protonated AsO_3_OH groups was postulated for lotharmeyerite by Kampf *et al.* (1984) from the infrared spectral measurement and by analogy also for ferrilotharmeyerite by Ansell *et al.* (1992). According to Ferraris & Ivaldi (1984), a protonated AsO_3_OH tetrahedron is generally distorted with the As—OH bond distance noticeably longer than the other three As—O bond distances. This appears to be the case for all monoclinic arsenate minerals in the natrochalcite-group, such as ferrilotharmeyerite, tsumcorite, mounanaite, gartrellite (Krause *et al.*, 1998), cobaltlotharmeyerite (Krause *et al.* 1999), nickellotharmeyerite (Krause *et al.*, 2001), and manganlotharmeyerite (Brugger *et al.*, 2002). In all these minerals, the As—O2 bond distance is the longest within the AsO_4_ group. However, from the crystal-chemical considerations, Krause *et al.* (1998) ruled out the likelihood for O2 being protonated, due to its coordination by one *A*, two *M*, and one *X* cations, which gives rise to a nearly ideal bond-valence sum (2.0 v.u.) for O2.  Moreover, Krause *et al.* (1998) argued that a protonated AsO_3_OH tetrahedron, in general, exhibits a decrease in the OH—As—O angles and an increase in the O—As—O angles, but they were unable to verify such a variation for the natrochalcite-group minerals. Our refinement on lotharmeyerite lends further support to the conclusion by Krause *et al.* (1998) that there is no evidence for the presence of the HAsO_4_ group in this structure. Specifically, the As—O bond lengths in lotharmeyerite vary from 1.671 (2) to 1.698 (2) Å, with an average of 1.688 Å. No outstanding long As—O bond is observed. Considering the experimental uncertainties, the difference between the longest As—O2 and next longest As—O3 bond distances is essentially insignificant [1.698 (2) Å *versus*. 1.692 (1) Å].  Furthermore, the O2—As—O3 and O2—As—O4 angles are 111.60 (6) and 101.87 (11)°, respectively, which are compared to the O3—As—O3 and O3—As—O4 angles of 108.93 (10) and 111.37 (7)°, respectively.  The calculated bond-valence sum for O2 in lotharmeyerite is 1.94 v.u.

Two distinct hydrogen bonds are present in lotharmeyerite, one between symmetry related O1 atoms [(x, y, z) and (1-z, y, 1-z)] and the other between O1 and O4 [at (x, y, z) and (0.5-x, 0.5+y, -z) respectively].  However, our refined H1 position is not half way between the two O1 atoms, indicating a non-centric, *i.e*. split position of the H1 atom.  Similar results have been observed in other natrochalcite-type minerals, such as natrochalcite NaCu_2_(SO_4_)_2_(OH).H_2_O (Chevrier *et al.*, 1993), cabalzerite CaMg_2_(AsO_4_)_2_.2H_2_O (Brugger *et al.* 2000), and synthetic Co- and Ni-analogs of natrochalcite (Krickl & Wildner, 2007). Such a hydrogen bonding scheme has also been discussed in detail by Tillmanns & Gebert (1973), Krause *et al.* (1998, 1999, 2001), and Brugger *et al.* (2002).

### Experimental

The lotharmeyerite crystal used in this study is from the type locality Mapimi, Durango, Mexico and is in the collection of the RRUFF project (deposition No. R060682; http://rruff.info).  The experimental chemical composition, Ca_0.99_(Zn_1.01_Mn^3+^_0.85_)(As_1.03_O_4_)_2_(H_2_O,OH)_2_, was determined with a CAMECA SX100 electron microprobe at the conditions of 15 kV, 10 nA, and a beam size of 5 µm (http//rruff.info).

### Refinement

A further empirical absorption correction for the X-ray intensity data was made using the program XABS2 (Parkin *et al.*, 1995), which significantly flattened the residual difference map features from 1.425 and -0.847 eÅ^-3^ to 0.808 and -0.767 eÅ^-3^ and lowered *R*_1_ to 1.88% from 2.24%. Two H atoms were located near O1 from difference Fourier syntheses and their positions refined freely with a fixed isotropic displacement (*U_iso_* = 0.04). The occupancy of the H1 site was fixed to 50% because of its splitting. During the structure refinements, for simplicity, we assumed the full occupations of the three non-hydrogen cation sites *A*, *M*, and *X* by Ca, (Zn + Mn), and As, respectively, with the Zn/Mn ratio refined. The resultant structural formula is Ca_1.00_(Zn_1.02_Mn^3+^_0.98_)(As_1.00_O_4_)_2_[(OH)_0.98_.1.04H_2_O]. The amount of OH is given for the charge balance. The highest residual peak in the difference Fourier maps was located at (0.3600, 0, 0.2766), 0.82 Å from O2, and the deepest hole at (0.4798, 0, 0.6433), 1.10 Å from As1.

### Crystal data

**Table:**

Ca(Zn·Mn)_2_(AsO_4_)_2_(H_2_O·OH)_2_	*F*(000) = 448
*M**_r_* = 474.14	*D*_x_ = 4.186 Mg m^−^^3^
Monoclinic, *C*2/*m*	Mo *K*α radiation, λ = 0.71073 Å
Hall symbol: -C 2y	Cell parameters from 1568 reflections
*a* = 9.0727 (6) Å	θ = 4.0–29.5°
*b* = 6.2530 (4) Å	µ = 14.38 mm^−^^1^
*c* = 7.4150 (5) Å	*T* = 293 K
β = 116.739 (4)°	Cube, brown
*V* = 375.68 (4) Å^3^	0.06 × 0.05 × 0.05 mm
*Z* = 2	

### Data collection

**Table:**

Bruker APEXII CCD area-detector diffractometer	739 independent reflections
Radiation source: fine-focus sealed tube	659 reflections with *I* > 2σ(*I*)
graphite	*R*_int_ = 0.022
φ and ω scan	θ_max_ = 32.6°, θ_min_ = 3.1°
Absorption correction: multi-scan [*SADABS* (Sheldrick, 2005) and *XABS2* (Parkin *et al.*, 1995)]	*h* = −13→13
*T*_min_ = 0.477, *T*_max_ = 0.532	*k* = −8→9
2512 measured reflections	*l* = −11→8

### Refinement

**Table:**

Refinement on *F*^2^	Secondary atom site location: difference Fourier map
Least-squares matrix: full	Hydrogen site location: difference Fourier map
*R*[*F*^2^ > 2σ(*F*^2^)] = 0.019	All H-atom parameters refined
*wR*(*F*^2^) = 0.045	*w* = 1/[σ^2^(*F*_o_^2^) + (0.030*P*)^2^] where *P* = (*F*_o_^2^ + 2*F*_c_^2^)/3
*S* = 0.91	(Δ/σ)_max_ < 0.001
739 reflections	Δρ_max_ = 0.81 e Å^−^^3^
49 parameters	Δρ_min_ = −0.77 e Å^−^^3^
0 restraints	Extinction correction: *SHELXL97* (Sheldrick, 2008), Fc^*^=kFc[1+0.001xFc^2^λ^3^/sin(2θ)]^-1/4^
Primary atom site location: structure-invariant direct methods	Extinction coefficient: 0

### Special details

**Table:**

Geometry. All e.s.d.'s (except the e.s.d. in the dihedral angle between two l.s. planes) are estimated using the full covariance matrix. The cell e.s.d.'s are taken into account individually in the estimation of e.s.d.'s in distances, angles and torsion angles; correlations between e.s.d.'s in cell parameters are only used when they are defined by crystal symmetry. An approximate (isotropic) treatment of cell e.s.d.'s is used for estimating e.s.d.'s involving l.s. planes.
Refinement. Refinement of *F*^2^ against ALL reflections. The weighted *R*-factor *wR* and goodness of fit *S* are based on *F*^2^, conventional *R*-factors *R* are based on *F*, with *F* set to zero for negative *F*^2^. The threshold expression of *F*^2^ > σ(*F*^2^) is used only for calculating *R*-factors(gt) *etc*. and is not relevant to the choice of reflections for refinement. *R*-factors based on *F*^2^ are statistically about twice as large as those based on *F*, and *R*- factors based on ALL data will be even larger.

### Fractional atomic coordinates and isotropic or equivalent isotropic displacement parameters (Å^2^)

**Table:**

	*x*	*y*	*z*	*U*_iso_*/*U*_eq_	Occ. (<1)
Ca1	0.0000	0.0000	0.0000	0.01360 (16)	
Zn1	0.2500	0.2500	0.5000	0.00936 (11)	0.512 (8)
Mn1	0.2500	0.2500	0.5000	0.00936 (11)	0.488 (8)
As1	0.41579 (3)	0.0000	0.20474 (4)	0.00864 (9)	
O1	0.3390 (3)	0.5000	0.4132 (3)	0.0140 (4)	
O2	0.3182 (3)	0.0000	0.3536 (3)	0.0153 (4)	
O3	0.03469 (18)	0.2798 (2)	0.2437 (2)	0.0127 (3)	
O4	0.2569 (3)	0.0000	−0.0265 (3)	0.0199 (5)	
H1	0.440 (9)	0.5000	0.464 (16)	0.040*	0.50
H2	0.298 (6)	0.5000	0.313 (7)	0.040*	

### Atomic displacement parameters (Å^2^)

**Table:**

	*U*^11^	*U*^22^	*U*^33^	*U*^12^	*U*^13^	*U*^23^
Ca1	0.0185 (4)	0.0120 (3)	0.0108 (4)	0.000	0.0070 (3)	0.000
Zn1	0.00931 (18)	0.00899 (16)	0.0080 (2)	0.00002 (10)	0.00232 (14)	−0.00002 (11)
Mn1	0.00931 (18)	0.00899 (16)	0.0080 (2)	0.00002 (10)	0.00232 (14)	−0.00002 (11)
As1	0.00787 (14)	0.00860 (12)	0.00895 (16)	0.000	0.00336 (11)	0.000
O1	0.0098 (10)	0.0201 (10)	0.0092 (11)	0.000	0.0017 (8)	0.000
O2	0.0192 (11)	0.0116 (8)	0.0220 (12)	0.000	0.0155 (10)	0.000
O3	0.0122 (7)	0.0110 (6)	0.0146 (8)	0.0023 (5)	0.0057 (6)	−0.0005 (5)
O4	0.0150 (11)	0.0294 (12)	0.0111 (11)	0.000	0.0020 (9)	0.000

### Geometric parameters (Å, °)

**Table:**

Ca1—O4^i^	2.426 (2)	Zn1—O1	1.9940 (14)
Ca1—O4	2.426 (2)	Zn1—O3^iv^	2.0303 (15)
Ca1—O3	2.4318 (14)	Zn1—O3	2.0303 (15)
Ca1—O3^i^	2.4318 (14)	Zn1—O2^iv^	2.1468 (14)
Ca1—O3^ii^	2.4318 (14)	Zn1—O2	2.1468 (14)
Ca1—O3^iii^	2.4318 (14)	As1—O4	1.671 (2)
Ca1—O2	2.898 (2)	As1—O3^v^	1.6918 (13)
Ca1—O2^i^	2.898 (2)	As1—O3^vi^	1.6918 (13)
Zn1—O1^iv^	1.9940 (14)	As1—O2	1.698 (2)
			
O4^i^—Ca1—O4	180.00 (10)	O3^ii^—Ca1—O2^i^	114.55 (4)
O4^i^—Ca1—O3	75.38 (5)	O3^iii^—Ca1—O2^i^	65.45 (4)
O4—Ca1—O3	104.62 (5)	O2—Ca1—O2^i^	180.00 (9)
O4^i^—Ca1—O3^i^	104.62 (5)	O1^iv^—Zn1—O1	180.0
O4—Ca1—O3^i^	75.38 (5)	O1^iv^—Zn1—O3^iv^	89.12 (7)
O3—Ca1—O3^i^	180.00 (7)	O1—Zn1—O3^iv^	90.88 (7)
O4^i^—Ca1—O3^ii^	75.38 (5)	O1^iv^—Zn1—O3	90.88 (7)
O4—Ca1—O3^ii^	104.62 (5)	O1—Zn1—O3	89.12 (7)
O3—Ca1—O3^ii^	92.03 (7)	O3^iv^—Zn1—O3	180.0
O3^i^—Ca1—O3^ii^	87.97 (7)	O1^iv^—Zn1—O2^iv^	99.04 (6)
O4^i^—Ca1—O3^iii^	104.62 (5)	O1—Zn1—O2^iv^	80.96 (6)
O4—Ca1—O3^iii^	75.38 (5)	O3^iv^—Zn1—O2^iv^	88.18 (7)
O3—Ca1—O3^iii^	87.97 (7)	O3—Zn1—O2^iv^	91.82 (7)
O3^i^—Ca1—O3^iii^	92.03 (7)	O1^iv^—Zn1—O2	80.96 (6)
O3^ii^—Ca1—O3^iii^	180.00 (12)	O1—Zn1—O2	99.04 (6)
O4^i^—Ca1—O2	121.94 (7)	O3^iv^—Zn1—O2	91.82 (7)
O4—Ca1—O2	58.06 (7)	O3—Zn1—O2	88.18 (7)
O3—Ca1—O2	65.45 (4)	O2^iv^—Zn1—O2	180.0
O3^i^—Ca1—O2	114.55 (4)	O4—As1—O3^v^	111.37 (7)
O3^ii^—Ca1—O2	65.45 (4)	O4—As1—O3^vi^	111.37 (7)
O3^iii^—Ca1—O2	114.55 (4)	O3^v^—As1—O3^vi^	108.93 (10)
O4^i^—Ca1—O2^i^	58.06 (7)	O4—As1—O2	101.87 (11)
O4—Ca1—O2^i^	121.94 (7)	O3^v^—As1—O2	111.60 (6)
O3—Ca1—O2^i^	114.55 (4)	O3^vi^—As1—O2	111.60 (6)
O3^i^—Ca1—O2^i^	65.45 (4)		

Symmetry codes: (i) −*x*, −*y*, −*z*; (ii) *x*, −*y*, *z*; (iii) −*x*, *y*, −*z*; (iv) −*x*+1/2, −*y*+1/2, −*z*+1; (v) *x*+1/2, −*y*+1/2, *z*; (vi) *x*+1/2, *y*−1/2, *z*.

### Hydrogen-bond geometry (Å, °)

**Table:**

*D*—H···*A*	*D*—H	H···*A*	*D*···*A*	*D*—H···*A*
O1—H1···O1^vii^	0.82 (7)	1.79 (8)	2.610 (4)	177 (11)
O1—H2···O4^viii^	0.66 (5)	1.95 (5)	2.595 (3)	163 (6)

Symmetry codes: (vii) −*x*+1, −*y*+1, −*z*+1; (viii) −*x*+1/2, −*y*+1/2, −*z*.
